# Compressive Strength Prediction of Rice Husk Ash Concrete Using a Hybrid Artificial Neural Network Model

**DOI:** 10.3390/ma16083135

**Published:** 2023-04-16

**Authors:** Chuanqi Li, Xiancheng Mei, Daniel Dias, Zhen Cui, Jian Zhou

**Affiliations:** 1 Laboratory 3SR, CNRS UMR 5521, Grenoble Alpes University, 38000 Grenoble, France; daniel.dias@univ-grenoble-alpes.fr; 2 Institute of Rock and Soil Mechanics, Chinese Academy of Sciences, Wuhan 430071, China; xcmei@whrsm.ac.cn (X.M.); zcui@whrsm.ac.cn (Z.C.); 3 School of Resources and Safety Engineering, Central South University, Changsha 410083, China

**Keywords:** rice husk ash, concrete, compressive strength, reptile search algorithm with circle mapping, artificial neural network

## Abstract

The combination of rice husk ash and common concrete both reduces carbon dioxide emission and solves the problem of agricultural waste disposal. However, the measurement of the compressive strength of rice husk ash concrete has become a new challenge. This paper proposes a novel hybrid artificial neural network model, optimized using a reptile search algorithm with circle mapping, to predict the compressive strength of RHA concrete. A total of 192 concrete data with 6 input parameters (age, cement, rice husk ash, super plasticizer, aggregate, and water) were utilized to train proposed model and compare its predictive performance with that of five other models. Four statistical indices were adopted to evaluate the predictive performance of all the developed models. The performance evaluation indicates that the proposed hybrid artificial neural network model achieved the most satisfactory prediction accuracy regarding R^2^ (0.9709), VAF (97.0911%), RMSE (3.4489), and MAE (2.6451). The proposed model also had better predictive accuracy than that of previously developed models on the same data. The sensitivity results show that age is the most important parameter for predicting the compressive strength of RHA concrete.

## 1. Introduction

Concrete is globally still one of the most highly demanded materials in construction and other industries [1]. By 2018, the production of concrete exceeded 10 billion cubic meters [2]. As a main component, the production of cement rose to 4 billion tons in 2020 [3]. Although cement provides the necessary strength for concrete, carbon dioxide (CO_2_) produced in the forging process is a heavy burden (approximately 7%) on the atmosphere. Considering the harm of CO_2_ to the environment and human beings, energy conservation and emission reduction have become normal goals in concrete application. Searching for ovel materials to replace parts of cement, namely, supplementary cementitious materials (SCMs), is one of the most effective ways to solve this problem.

Most available SCM options are derived from byproducts associated with industrial and agricultural processes, such as palm-oil fuel [4,5], olive-oil [6,7], and fly [8,9] ash, silica fume [10,11], seed shells [12], dispersed coconut fibers [13], and other types of powder [14,15,16,17,18,19,20]. Among these novel SCMs, the combination of rick husk ash (RHA) and conventional concrete has received much attention [21,22,23]. First, RHA is one of the main byproducts of agricultural production. Conventional stacking could pollute the air and groundwater [24], but adding it to concrete is a reasonable and innovative way to recycle. Second, the pozzolanic nature of RHA helps in improving the durability and strength of concrete [25]. However, the addition of RHA has an important effect on concrete performance [26], especially compressive strength, which directly affects the durability and stability of structures in construction and other industries. Madandoust et al. [27] used RHA to replace 20% of cement to study the strength of concrete. Their results showed that the short-term compressive strength of RHA concrete was reduced, but the long-term compressive strength was increased. Ahsan and Hossain [28] compared cement performance at different RHA replacement rates (10% and 20%). They found that replacing 10% of cement with RHA was optimal because the interfacial transition zone was more effectively densified with the silica content of RHA. However, Noaman et al. [29] reported that that replacing cement with 15% RHA could maximize concrete’s compressive strength. Furthermore, determining the mixing ratio of other components in concrete production with cement and RHA is complicated; thus, it is both necessary and challenging to determine the strength of concrete.

The most accurate strength measurement method of concrete is the compressive test in the laboratory. However, the production and maintenance of concrete samples is complicated and time-consuming, and wastes workers and material resources [30]. For example, a group of experiments require two to three professionals to complete. In order to improve calculation efficiency and site limitation, a method based on an empirical formula was developed to estimate compressive strength that was especially praised by field workers. Islam et al. [31] developed an empirical formular by using the least-squares approach to calculate the compressive strength of RHA concrete. Their results showed that the formular achieved good predictive performance with a correlation coefficient (R) of 0.816. Liu et al. [32] utilized six empirical equations to estimate the compressive strength of concrete containing RHA with different replacement values. However, the limitation of the empirical formula is that it cannot accurately express the complex nonlinear relationship between the considered parameters and compressive strength [33].

In recent years, artificial-intelligence methods with machine learning (ML) as a mainstream technologies have been widely used to solve the problem of concrete strength prediction [34,35,36,37,38,39,40]. Azimi-Pour et al. [41] utilized four types of support vector machine (SVM) models to predict the compressive strength of fly ash concrete. The performance results indicated that the radial basis function (RBF)-based SVM model had the highest accuracy for a coefficient of determination (R^2^) equal to 0.9932. Zhang et al. [42] improved the random forest (RF) model to predict the compressive strength of lightweight concrete (LWC). The extreme learning machine (ELM) model was applied for the compressive strength prediction of lightweight foamed concrete [43]. Compared with these models, an artificial neural network (ANN) model with a simple structure, and good capabilities for processing high-dimensional data and complex parameter relationships is more favored in predicting the concrete strength of RHA [44,45,46,47]. Getahun et al. [48] developed an ANN model to forecast the 28-day compressive strength of a composite concrete mixture with RHA and reclaimed asphalt pavement (RAP). The prediction results illustrated that the ANN model could accurately fit the relationship between the considered components and the strength, as evidenced by excellent performance indices: R was 0.9811 and the root mean square error (RMSE) was 0.648. To optimize the selection scheme of the ANN model on weight and bias values, and further improve model performance, many scholars modified this model using numerous optimization algorithms for predicting concrete strength, e.g., grey wolf optimization (GWO) [49,50], particle swarm optimization (PSO) [51,52], the genetic algorithm (GA) [53], the whale optimization algorithm (WOA) [54], and simulated annealing (SA) with PSO [55]. For the strength prediction of RHA concrete, Andalib et al. [56] utilized the bat algorithm (BA), PSO, and teaching–learning-based optimization (TLBO) algorithm to optimize the ANN model for predicting compressive strength. The performance results showed that all optimized ANN models achieved satisfactory prediction accuracy, especially the BA–ANN model (RMSE = 5.898); Hamidian et al. [33] proposed four hybrid ANN models to estimate the compressive strength of RHA concrete. On the basis of the results of the performance analysis of all models, the PSO-with-two differential-mutations (PSOTD)-based ANN model achieved superior performance than that of other models, indicated by the higher R^2^ values (0.9697). There are still many newly developed and excellent optimization-algorithm-based populations that have not been applied to the strength prediction of RHA concrete. Population initialization also needs attention to maximize the predictive potential of ANN models.

Therefore, this paper utilizes circle mapping (CM) to improve the optimization performance of the reptile search algorithm (RSA). A novel hybrid ANN model optimized with CMRSA is proposed to estimate the compressive strength of RHA concrete. The predictive accuracy of four ML models and an empirical model was compared. These ML models consisted of optimized and common models: seagull optimization algorithm (SOA)-based SVM (SOA–SVM) and RF (SOA–RF) models, an ANN model, and an ELM model. Four statistical indices, regression analysis, error comparison, and the Taylor diagram were adopted to evaluate the predictive performance of all models in order to determine the optimal model. Lastly, sensitivity analysis was performed to select the most important parameter for predicting the compressive strength of RHA concrete.

## 2. Data and Methods

### 2.1. Rice Husk Ash Concrete

RHA concrete cannot be produced without the use of other materials. For example, cement is used to provide sufficient strength for concrete, water is key to controlling concrete compactness in the mixing process, and the aggregate maintains concrete volume stability. To assess the compressive strength of RHA concrete, Iftikhar et al. [57] combined cement (kg/m^3^), RHA (kg/m^3^), a superplasticizer (kg/m^3^), an aggregate (kg/m^3^), and water (kg/m^3^) to produce a series of concrete samples. Freshly poured concrete needs to be cured, and its strength must be measured after a certain time. Therefore, age (days) is also an important variable in predicting concrete strength. In this paper, 192 compressive-strength data from Iftikhar et al. [57] were utilized to evaluate RHA concrete. The statistical information of these variables and the compressive strength of the target concrete samples is listed in Table 1.

For establishing the prediction model, all variables except compressive strength were taken as the input parameters. The interdependence of the input parameters must be evaluated to simplify the model and maintain prediction accuracy. The correlation coefficient is widely used to describe dependence [58,59,60]. If the correlation coefficient between any two input parameters exceeds 0.8, parameter deletion should be considered. Table 2 shows the calculation results of correlation coefficient values between input parameters. The maximal correlation coefficient value was 0.549, induced by water and the aggregate. Therefore, all input parameters could be considered for generating a prediction model for estimating the compressive strength of RHA concrete.

### 2.2. Reptile Search Algorithm

RSA is a novel metaheuristic optimization-algorithm-based algorithm proposed by Abualigah et al. [61]. This algorithm was inspired by the hunting behavior of crocodiles to solve the optimization problem. As the apex predators in amphibious environments, the behavior of crocodiles has long attracted the attention of scientists. Crocodiles are highly mobile, and can thereby quickly chase and attack prey, especially at night. The crocodile’s excellent night vision and body shape with little resistance benefit this feature [61]. Second, crocodiles are highly intelligent animals, which endows them with high recognition and high perception capabilities. For instance, crocodiles wait where prey is frequent, such as near a river. Crocodile hunting is also group behavior, and teams with a clear division of labor enable individuals to obtain enough food. Therefore, the first step in performing a hunting campaign is to initialize the population in the search space as follows:

(1) $$C_{ij}=rand\cdot (U_{b}-L_{b})+L_{b}$$

where $C_{ij}$ represents the *j*-th position of the *i*-th crocodile; $U_{b}$ and $L_{b}$ represent the upper and lower bounds of the search space, respectively; *rand* is a random number. The setting of *rand* indicates that the individual position is randomly determined to find the prey. However, population diversity and the possible search area are limited by this random initialization-method-based mechanism [62]. To solve this problem, various types of chaos mapping were combined to establish the different distributions of individuals in the search space [63,64]. In this paper, circle mapping, with the advantages of stability and coverage rate, was utilized to optimize the population initialization of RSA.

(2) $$C_{ij}=C_{ij}+H-\left(\frac{G}{2\pi }\right)sin\left(2\pi C_{ij}\right)mod(1)$$

where *H* and *G* represent the externally applied frequency and strength of nonlinearity, respectively.

**Figure 1:**
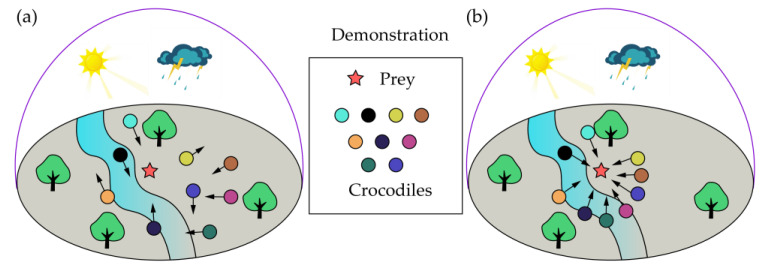
Illustration of hunting behavior for RSA proposed by Abualigah et al. [61]: (**a**) exploration; (**b**) exploitation.

After determining the initial positions of individuals, the exploration command was executed to find and encircle prey in the search space (see Figure 1a). In this phase, two strategies could be selected by the crocodiles to search the entire area as much as possible. The mathematical expressions of these strategies are as follows:

(3) $$C_{ij}^{t+1}=\left\{\begin{array}{l} Best^{t}-\partial_{ij}^{t}\cdot \alpha -F_{ij}^{t}\cdot rand,\;\;\;t\leq \frac{T}{4}\\ Best^{t}\cdot C_{ij}\cdot \eta^{t}\cdot rand,\;\;\;t\leq \frac{T}{2}\;\mathrm{and}\;t>\frac{T}{4} \end{array}\right.$$

where $C_{ij}^{t+1}$ represents the *j*-th position of the *i*-th crocodile at the *t* + 1 iteration; *T* is the maximal iteration value; $Best^{t}$ indicates the best position at the current (*t*) iteration, $\partial_{ij}^{t}$ represents an internal parameter, namely, the hunting operator for the *j*-th position of the *i*-th crocodile at the current iteration; α represents a related parameter to exploration accuracy, which was equal to 0.1 in this paper; $F_{ij}^{t}$ and $\eta^{t}$ are the reduce function and evolutionary sense, respectively. The former is used to narrow the search in a limited space, and the latter is a probability ratio.

Once the prey is encircled by crocodiles, the hunting (i.e., exploitation, as shown in Figure 1b) can be performed, which uses two strategies, coordination and cooperation, to determine the optimal crocodile position. Two strategies in this phase are mathematically expressed as follows:

(4) $$C_{ij}^{t+1}=\left\{\begin{array}{l} Best^{t}\cdot P_{ij}^{t}\cdot rand,\;\;\;t\leq \frac{3T}{4}\;\mathrm{and}\;t>\frac{T}{2}\\ Best^{t}-\partial_{ij}^{t}\cdot \mu -F_{ij}^{t}\cdot rand,\;\;\;t\leq T\;\mathrm{and}\;t>\frac{3T}{4} \end{array}\right.$$

where $P_{ij}^{t}$ represents the percentage difference between the best and current positions, and μ is a small value in RSA. In general, the aim of the combination of coordination and cooperation is to avoid falling into local optima.

## 3. Development of the Novel CMRSA–ANN Model

**Figure 2:**
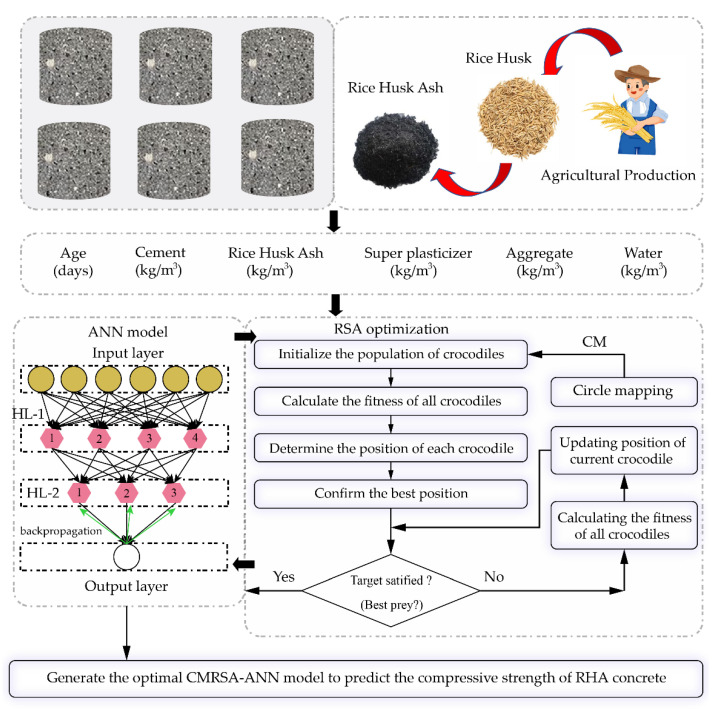
Flowchart of generating CMRSA–ANN model to predict the compressive strength of RHA concrete.

In this paper, the ANN model was generated to accurately predict the strength of RHA concrete. However, the design of an ANN structure has an important effect on predictive performance. In particular, the determination of weights and biases among the input, hidden, and output layers is difficult and challenging [65]. The improved RSA using Circle mapping (CM) was utilized to find the optimal weights and biases for the ANN model. To that end, a novel prediction model, CMRSA–ANN model, was proposed to predict the compressive strength of RHA concrete. Before running the model, a total of 192 data were randomly divided into training and test sets at a 4 to 1 ratio, i.e., 154 data were utilized to train the model, and 38 data to verify the model performance. All data can be found in the Supplementary materials. Since the units of all used parameters were different, the necessary normalization could avoid this impact on performance development. Thus, all parameters were normalized in the range from −1 to 1. For the optimization-algorithm-based population, population size is the most important internal parameter that needs to be determined during iterations [66,67,68]. To find the global optimal solution, six population sizes (25, 50, 75, 100, 125, and 150) were adopted to conduct the optimization process for the ANN model. We set up 300 iterations to ensure that the optimal solution could be found and remain stable. In general, the fitness value was utilized to represent the solution calculated with the optimization algorithm. In this paper, RMSE was used to generate a fitness function for evaluating optimization performance. The flowchart of developing CMRSA–ANN models to predict the compressive strength of RHA concrete is shown in Figure 2.

Other models (SOA–SVM, SOA–RF, ANN, ELM, and an empirical formular) were also developed to predict concrete strength, and their prediction results were compared with those of the CMRSA–ANN model. To select the best prediction model, four statistical indices were considered to evaluate the predictive performance of each model: R^2^, RMSE, variance accounted for (VAF), and mean absolute error (MAE). The definition of these indices can be found in the literature [69,70], and their formulars are expressed as follows:

(5) $$R^{2}=1-\frac{{\left[\sum_{t=1}^{T}\left(C_{t}-c_{t}\right)\right]}^{2}}{{\left[\sum_{t=1}^{T}\left(C_{t}-\bar{C}\right)\right]}^{2}}$$

(6) $$\mathrm{VAF}=\left[1-\frac{\mathrm{var}(C_{t}-c_{t})}{\mathrm{var}(C_{t})}\right]\times 100$$

(7) $$\mathrm{RMSE}=\sqrt{\frac{1}{T}\sum_{t=1}^{T}{\left(C_{t}-c_{t}\right)}^{2}}$$

(8) $$\mathrm{MAE}=\frac{1}{T}\sum_{t=1}^{T}\left|C_{t}-c_{t}\right|$$

where *T* is the maximal number of samples; *C_t_* and *c_t_* are the values of the *t*-th measured and predicted, respectively; $\bar{C}$ is the average of the measured values.

## 4. Prediction Model Development

Before applying the ideal proposed model to predict the compressive strength of RHA concrete, all models were developed using the same training set (80% of the database). The detailed development process of each model is shown in this section.

### 4.1. ANN Model

For a common ANN model, the basic structure is composed of input, hidden, and output layers. Compared with the number of hidden layers, one input layer and one output layer are fixed collocations in single-target regression tasks. Two hidden layers are often utilized to solve similar prediction problems [71,72,73]. Furthermore, the number of neurons in each hidden layer greatly impacts ANN model performance. Hence, a series of tests were carried to select the suitable ANN structure and the corresponding neurons for predicting the compressive strength of RHA concrete. In this paper, the hidden layers were 1 or 2, the range of neuron numbers was from 2 to 12, the activation function was set to sigmoid, and the backpropagation algorithm was utilized to improve the prediction accuracy. As a result, 10 tests with different ANN models were established, and their performance was represented by using R^2^ and RMSE, as shown in Table 3. The ANN model with two hidden layers (four neurons in the first hidden layer and three neurons in the second hidden layer) had the best performance, with a higher R^2^ (0.8772) and lower RMSE (5.8632) than those of other models.

### 4.2. CMRSA–ANN Model

**Figure 3:**
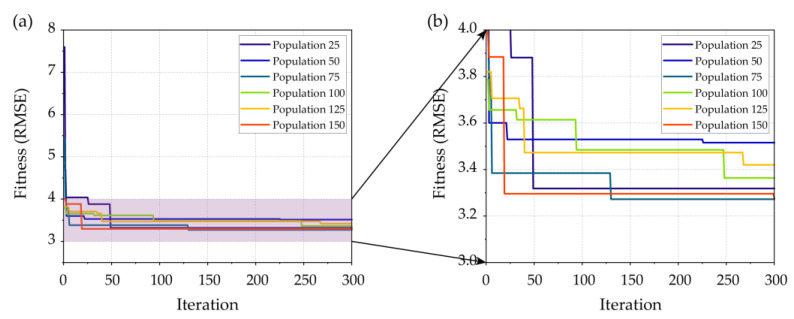
CMRSA–ANN model development: (**a**) iteration curves; (**b**) local comparison.

Although the best structure was determined in the ANN model development, it is difficult to choose weights and biases between layers to minimize prediction error. Therefore, the CMRSA optimization algorithm was utilized to optimize the initial ANN model with two hidden layers (four neurons in the first hidden layer and three neurons in the second hidden layer); the framework is shown in Figure 2. Six hybrid CMRSA–ANN models with different population sizes were run for 300 iterations. The iteration curve of each model is shown in Figure 3a. Figure 3b shows that the CMRSA–ANN model with a population size of 75 had the lowest fitness value among all hybrid ANN models. As a result, this model was used to predict the compressive strength of RHA concrete in this paper.

### 4.3. SOA–SVM Model

**Figure 4:**
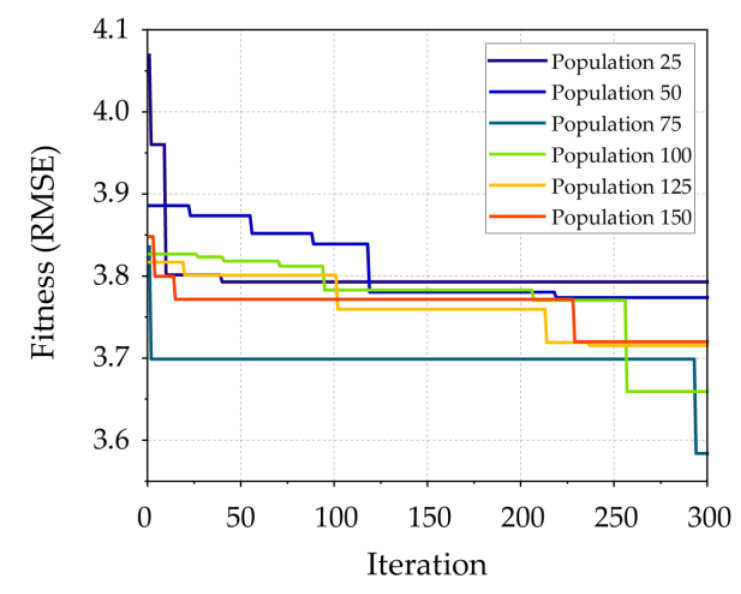
SOA–SVM model development.

The development of the SOA–SVM model is similar to that of the CMRSA–ANN model. For the SVM model, two main hyperparameters, the regularization parameter (*C*) and kernel coefficient (γ) of the used kernel function, are key players to improving the model performance [74,75]. In this paper, the popular radial basis function (RBF) was considered as the kernel function of the SVM model. To determine the optimal hyperparameter combination of the SVM model, the range of these parameters was 0 to 100. Thew population sizes and iteration number of SOA were set to be the same as those of the CMRSA. The development results of the SOA–SVM models are shown in Figure 4. The best SOA–SVM model had a population of 75 in the training phase and had a lower fitness value of RMSE than that of other models.

### 4.4. SOA–RF Model

**Figure 5:**
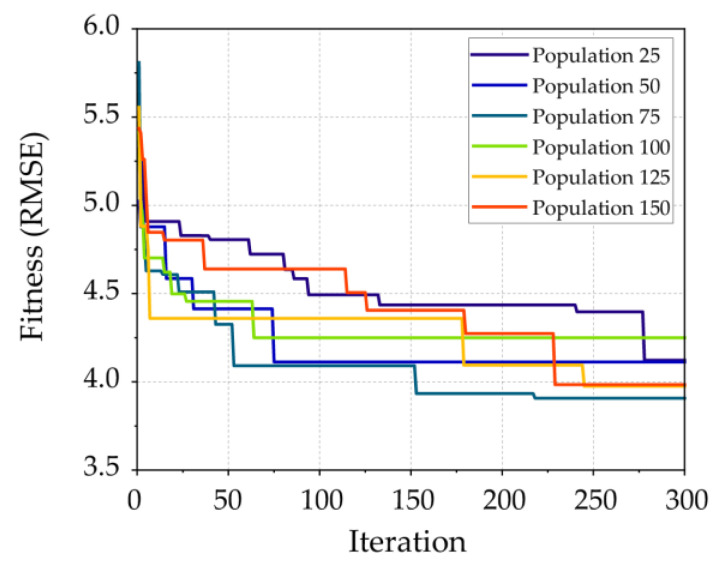
SOA–RF model development.

Ensemble models such as the RF model could achieve good performance in solving classification and regression problems; a detailed introduction of the RF model can be found in the literature [58,76]. The unique tree structure and bootstrap sampling allow for the RF performance to be determined by all trees and resist overfitting [74]. In the development of SOA–RF models, the main purpose is to find the best hyperparameter combination of the RF model, i.e., the number of tress (*Nt*) and the random features (*Maxdepth*). In this paper, the tree-number range was from 1 to 100, and the random-feature range was from 1 to 10. Figure 5 shows the optimization results of thew SOA–RF models based on different population sizes after 300 iterations. The OA–RF model containing a population of 75 achieved the most satisfactory performance, as shown by having the lowest fitness value of RMSE. Therefore, this SOA–RF model was considered to predict the compressive strength of RHA concrete.

### 4.5. ELM Model

The ELM model is a special neuron network with a single hidden layer for solving regression problems. The predictive performance of the ELM model is only controlled by the selection of neuron numbers in the hidden layer. To that end, 10 ELM models with various neuron numbers in the hidden layer were generated to predict concrete strength. Table 4 lists the predictive performance of each ELM model in the training phase. ELM models with large neuron numbers achieved better performance than that of models with smaller neuron numbers. However, the best ELM model was in the 9th test, when the neurons were 100. The performance indices of this model were more reliable than those of other models, i.e., R^2^ is equal to 0.8932 and RMSE is equal to 5.4682.

### 4.6. Empirical Model

**Figure 6:**
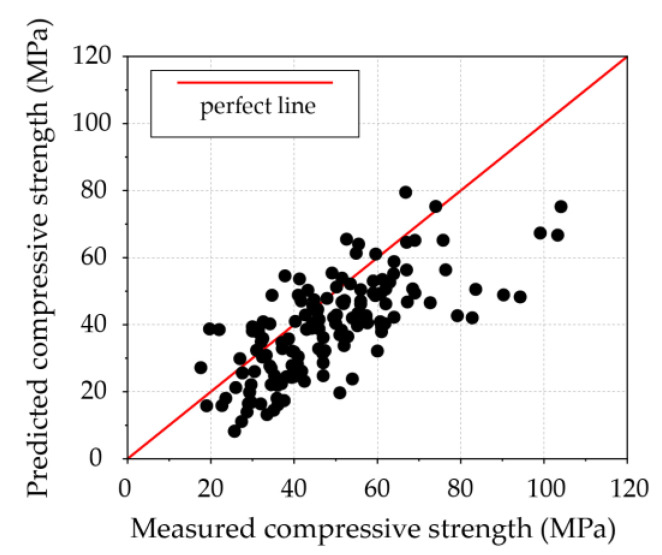
Performance of the empirical model using the training set.

The empirical model is an effective method that uses the relevant parameters to quickly achieve the target calculations. In this paper, six input parameters were considered into the empirical formular using multivariate linear regression, as expressed in Equation (9). The training performance of the developed empirical model is shown in Figure 6.

(9) Y=−0.47317 + 0.297X1 + 0.0779X2 − 0.0732X3 − 0.145X4 + 1.524X5 + 0.0154X6

where Y represents the compressive strength. X1–X6 represent the age, cement, RHA, water, superplasticizer, and aggregate, respectively.

## 5. Results and Discussion

**Figure 7:**
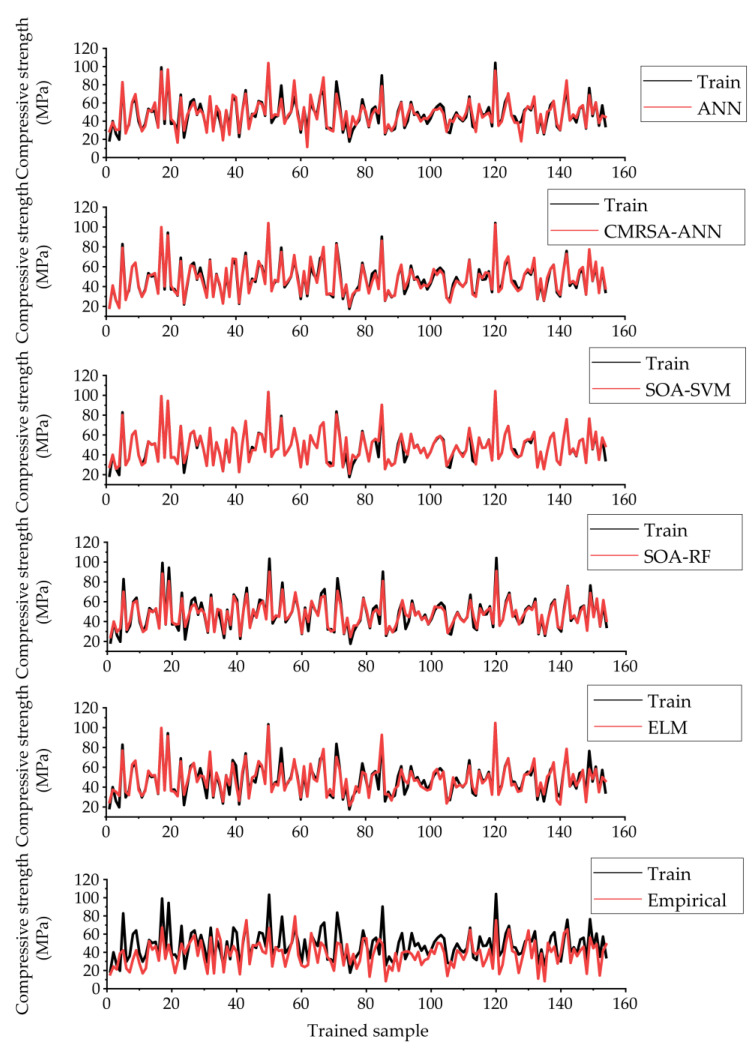
Prediction curves of the developed models using the training set.

After training the proposed models, the predictive performance of each model was properly evaluated. Figure 7 shows the prediction curves of all models for estimating the compressive strength of RHA concrete in the training phase. The difference between the prediction curve of the empirical model and the training curve was the greatest among all models. The similarity between the prediction curves of three hybrid models and training was relatively higher, especially in the CMRSA–ANN model.

However, all trained models needed to be further tested to ensure the retention of the excellent predictive ability. Table 5 illustrates the evaluation results of each model using four performance indices in both the training and the testing phases. The performance results from using the training set show that the CMRSA–ANN model was the best prediction model, as it had the highest values of R^2^ and VAF (0.9679 and 96.7884%), and the lowest values of RMSE and MAE (2.9991 and 2.3169). Following this model, two other hybrid models (SOA–SVM and SOA–RF) also had superior predictive accuracy than that of the unoptimized ML (ANN and ELM) and empirical models. On the other hand, the proposed CMRSA–ANN model still achieved better predictive performance than that of other models, indicated by the higher values of R^2^ and VAF (0.9709 and 97.0911%), and the lower values of RMSE and MAE (3.4489 and 2.6451). Although the performance of the SOA–SVM and SOA–RF models in the testing phase was worse than that using the training set, they still achieved higher predictive accuracy than that of the unoptimized ML models. The ANN model achieved better performance than that of the ELM model in the testing phase, proving that the prediction accuracy of the ELM model is unstable for solving regression problems.

**Figure 8:**
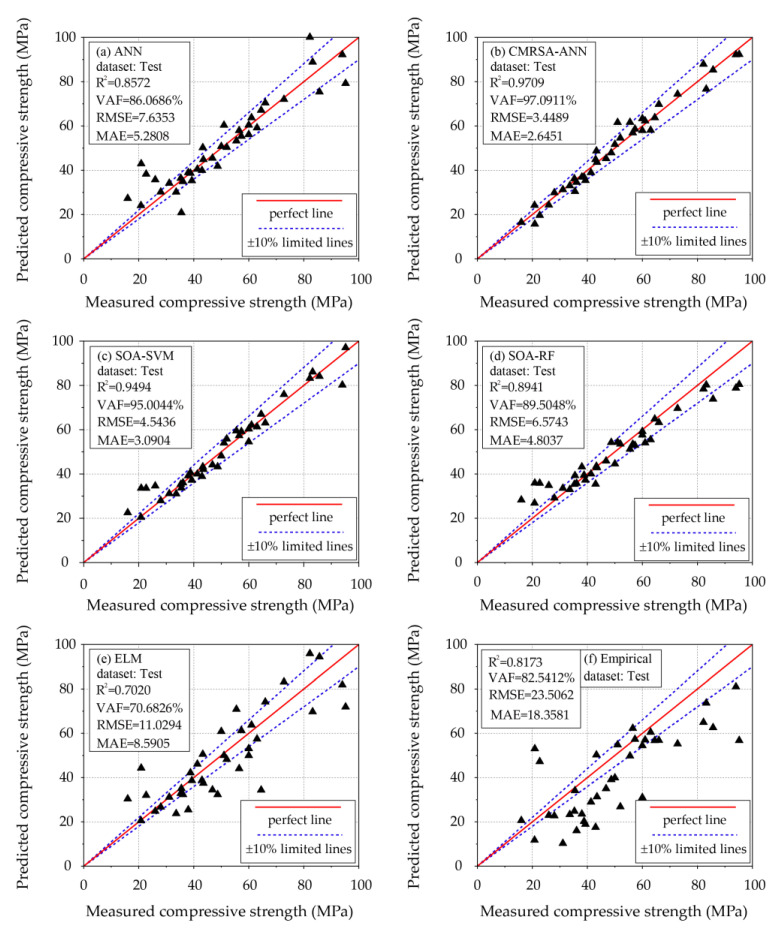
Regression results of all developed models using the test set.

Regression relationships can also be used to evaluate and compare the predictive performance of models. Figure 8 shows the regression results of each model using the test set. In each regression plot, one perfect and two limited lines were used to evaluate the regression relationship between the values from the prediction model and the measured values. For instance, the data point determined by the best model with a prediction accuracy of 100% could lie on the perfect line. Observations based on this criterion show that the CMRSA–ANN model achieved better predictive performance than that of other models, indicated by the greater number of data points close to the perfect line and within the limited lines. Furthermore, the performance of the SOA–SVM and SOA–RF models was better than that of the ANN, ELM, and empirical models, but they could not perform accurate predictions for small values of compressive strength (less than 30 MPa).

**Figure 9:**
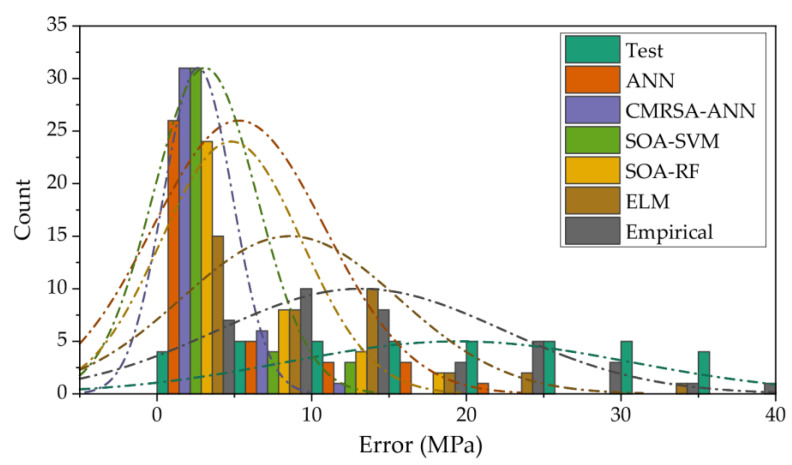
Error distribution of each prediction model in the testing phase.

For the regression problem, the error between the predicted and measured values was one of the most concerning performance indices. Although perfect predictions rarely exist, one of the purposes of training and testing is to shrink the prediction error of each target as much as possible. The measured and predicted values of the compressive strength of RHA concrete are listed in Table 6. Figure 9 illustrates the error distribution of each prediction model in the testing phase. The error by the CMRSA–ANN model was mainly concentrated within 10 MPa and accounted for the highest proportion within 5 MPa. The error distribution of the SOA–SVM model was similar to that of the CMRSA–ANN model in a small range where the error was less than 10 MPa, while there were some larger errors between 10 and 15 MPa. The error distribution from the empirical model was undoubtedly the most unsatisfactory, as it both accounted for the lowest proportion of small errors and had many excessive errors.

**Figure 10:**
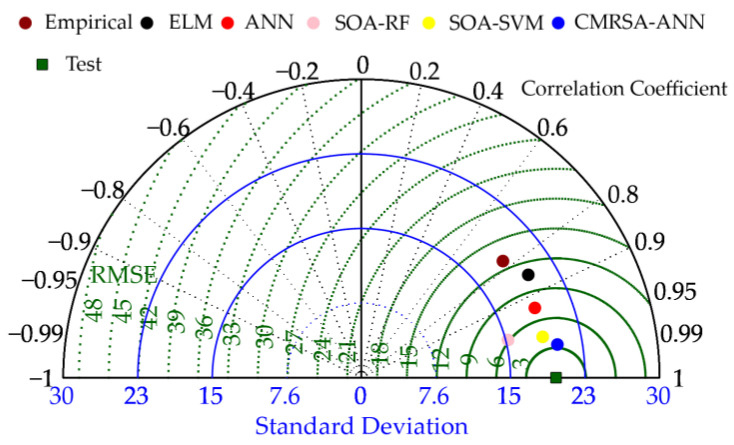
Performance comparison between prediction models using a Taylor diagram.

Taylor diagrams are used in visually comparing the predictive performance of multiple models. In a Taylor diagram, a model with high prediction accuracy is close to the position of the target value. The position of each model is determined with three indices, i.e., St. D., RMSE, and R. Therefore, the model performance can be evaluated and compared with multiple indices. Figure 10 displays the evaluation results of all developed models in the Taylor diagram. The CMRSA–ANN model was the closest to the position of the test set. Following this model, models sorted by distance are SOA–SVM, SOA–RF, ANN, ELM, and empirical. These results indicate that the CMRSA optimization algorithm is successful in improving the predictive performance of ANN models. The optimized SVM and RF models had better predictive accuracy than that of the unoptimized ANN and ELM models. Therefore, it is feasible to use the hybrid optimization model to predict the compressive strength of RHA concrete. CMRSA–ANN was selected as the optimal model in this paper.

**Figure 11:**
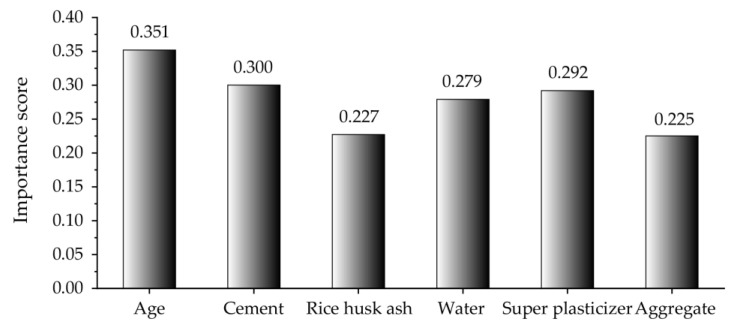
Importance score of each parameter based on the CMRSA-ANN model.

Nevertheless, the importance or sensitivity of each input parameter to the prediction of compressive strength is unknown, which is detrimental to further improving concrete properties. Therefore, sensitivity analysis was conducted to evaluate the impact of each input parameter on the output. In this paper, calculation method PAWN, proposed by Pianosi and Wagener [77,78], was adopted to calculate the importance score of the input parameters. Figure 11 illustrates the sensitivity results of the compressive strength prediction of the CMRSA–ANN model. Age was the most important parameter, with the highest score (0.351), for predicting the compressive strength of RHA concrete. After age, parameters ranked by influence are cement (0.300), the superplasticizer (0.292), water (0.279), RHA (0.227), and the aggregate (0.225). This result is consistent with that obtained by Iftikhar et al. [57].

In order to verify the effectiveness and superiority of the prediction model, the predictive performance of the other models developed using the same database was compared with that of the CMRSA–ANN model proposed in this paper, and the results are shown in Table 7. The proposed model had superior predictive performance than that of the published models, indicated by the higher R^2^ value. These results also indicate that the CMRSA–ANN model could better explain the relationship between the input parameters and the compressive strength of RHA concrete.

## 6. Conclusions

The combination of RHA and concrete not only solves the problem of carbon dioxide emissions from cement production and reduces the pressure of waste accumulation, but could also be widely used as a green building material. To evaluate the performance of RHA concrete, we proposed a novel hybrid CMRSA–ANN model to predict the compressive strength of RHA concrete. We utilized 192 concrete data to train the model and test its performance. Furthermore, four ML models and an empirical model were developed, and their prediction results were compared with those of the proposed model. The main conclusions of this paper are as follows:

(1) The proposed hybrid CMRSA–ANN model achieved the best prediction accuracy for R^2^ (0.9679 and 0.9709), VAF (96.7884% and 97.0911%), RMSE (2.9991 and 3.4489), and MAE (2.3169 and 2.6451) among all models in the both the training and the testing phases. The performance comparison between the proposed and optimized ANN models also indicated that the CMRSA could effectively improve the prediction ability of the ANN model.

(2) The empirical model could not better explain the relationship between the input parameters and the compressive strength of RHA concrete. Therefore, the empirical model was not suitable as a conventional means to evaluate concrete performance. 

(3) The hybrid SOA–SVM and SOA–RF models achieved better performance than that of the unoptimized ANN and ELM models, indicated by a higher R^2^ (0.9491 and 0.8941) and VAF (95.0044% and 89.5048%), and lower RMSE (4.5436 and 6.5743) and MAE (3.0904 and 4.8037) in the testing phase. It is effective and necessary to use an optimization (such as population-based) algorithm to improve the performance of ML models.

(4) Age was the most important input parameter for predicting the compressive strength of RHA concrete. However, other input parameters with similar importance scores should also be given high priority.

The purpose of this paper was to propose a new method for predicting RHA concrete strength, and the mining of the potential relationship among the data themselves through hybrid algorithm combination and optimization. However, the limitation of this paper is that the amount of data used for training and testing the models was always insufficient. An increase in effective data could help in improving the ability of the model to learn the potential relationship between input and output parameters, and the diversification of the test data could better verify the model performance. Therefore, adding more experimental data is an effective way to further improve the prediction accuracy of the model. Combinations of other optimization algorithms and different ML models in the performance prediction of RHA concrete are also worth comparing.

## Figures and Tables

**Table 1:** Statistical information on each variable for predicting RHA concrete strength.

Variables	Statistical Indices				
	Min	Max	Mean	Median	St. D
Cement	249.0	783.0	409.02	400.00	105.47
RHA	0.0	171.0	62.33	57.00	41.55
Superplasticizer	0.0	11.3	3.34	1.85	3.52
Aggregate	1040.0	1970.0	1621.51	1725.00	267.77
Water	120.0	238.0	193.54	203.00	31.93
Age	1.0	90.0	34.57	28.00	33.52
Compressive strength	16.0	104.1	48.14	45.95	17.54

Note: Min, minimal values; Max, maximal values; St. D, standard deviation.

**Table 2:** Correlation coefficient values between all considered parameters.

Variables	Cement	RHA	Superplasticizer	Aggregate	Water	Age	Compressive Strength
Cement	1	−0.219	0.253	−0.238	0.083	−0.106	0.370
RHA		1	−0.021	−0.139	0.136	−0.033	−0.023
Superplasticizer			1	−0.205	0.268	−0.000	0.301
Aggregate				1	−0.549	−0.063	0.147
Water					1	0.011	−0.244
Age						1	0.495
Compressive strength							1

**Table 3:** Performance of the ANN model with different hidden layers and neuron numbers.

Tests	Structure		Performance	
	HL-1	HL-2	R^2^	RMSE
1	2	/	0.8322	6.8525
2	4	/	0.7839	7.7690
3	6	/	0.8100	7.2921
4	8	/	0.8225	7.0476
5	10	/	0.8554	6.3611
6	4	3	0.8772	5.8632
7	4	6	0.8312	6.8726
8	6	8	0.8025	7.4350
9	8	10	0.8143	7.2101
10	10	12	0.8338	6.8193

Note: HL-1, first hidden layer; HL-2, second hidden layer.

**Table 4:** Performance of an ELM model with different neuron numbers.

Tests	Neuron Numbers	Performance	
		R^2^	RMSE
1	20	0.5268	11.5078
2	30	0.6460	9.9534
3	40	0.7327	8.6492
4	50	0.7595	8.2046
5	60	0.7851	7.7555
6	70	0.7997	7.4873
7	80	0.8589	6.2835
8	90	0.8373	6.7479
9	100	0.8932	5.4682
10	110	0.8788	5.8235

**Table 5:** Performance evaluation of prediction models using training and test sets.

Model	Performance (Training Set)				Model	Performance (Test Set)			
	R^2^	VAF %	RMSE	MAE		R^2^	VAF %	RMSE	MAE
ANN	0.8772	87.7619	5.8632	4.1423	ANN	0.8572	86.0686	7.6353	5.2808
CMRSA–ANN	0.9679	96.7884	2.9991	2.3169	CMRSA–ANN	0.9709	97.0911	3.4489	2.6451
SOA–SVM	0.9595	96.0957	3.3651	1.2528	SOA–SVM	0.9494	95.0044	4.5436	3.0904
SOA–RF	0.9224	92.2384	4.6610	3.2359	SOA–RF	0.8941	89.5048	6.5743	4.8037
ELM	0.8932	89.3163	5.4682	4.0644	ELM	0.7020	70.6826	11.0294	8.5905
Empirical	0.2023	50.0783	14.9418	12.0202	Empirical	0.3716	57.3263	16.0169	13.1709

**Table 6:** Prediction results of the compressive strength of RHA concrete using the developed models.

No.	Measured	Predicted					
		ANN	CMRSA–ANN	SOA–SVM	SOA–RF	ELM	Empirical
1	82.20	100.09	87.90	83.20	78.36	95.91	64.96
2	72.80	72.04	74.38	75.93	69.61	83.15	55.26
3	43.50	44.85	43.59	42.07	42.81	37.48	31.36
4	48.70	41.82	47.91	43.25	54.26	32.36	39.11
5	16.00	27.32	16.50	22.53	28.28	30.40	20.62
6	85.70	75.39	85.31	84.08	73.81	94.46	62.58
7	43.00	39.93	44.88	38.92	35.43	38.80	17.57
8	33.60	30.16	33.05	31.06	33.00	23.74	23.33
9	94.00	92.21	92.18	80.18	78.79	81.86	81.00
10	31.10	34.15	31.28	31.15	33.57	31.17	10.31
11	57.30	55.35	58.61	59.18	52.92	61.25	57.31
12	41.30	40.49	38.96	39.98	40.03	46.12	28.98
13	20.80	24.08	24.19	20.48	26.86	20.58	11.78
14	22.70	38.28	19.66	33.55	35.84	32.02	47.24
15	38.80	38.68	36.91	40.64	39.48	42.21	20.21
16	60.00	60.42	63.35	54.54	59.20	49.98	54.46
17	55.50	53.28	61.66	59.50	51.22	70.86	49.78
18	61.00	63.75	62.30	62.09	54.07	63.77	57.04
19	63.00	59.20	58.12	61.35	55.48	57.46	60.53
20	66.00	70.44	69.78	63.07	63.24	74.16	56.84
21	52.00	50.39	54.58	55.85	53.52	48.25	26.83
22	43.30	50.25	48.77	43.25	43.49	50.56	50.28
23	26.00	35.75	24.35	34.61	34.82	24.83	22.97
24	64.50	67.10	63.77	66.99	64.85	34.38	56.76
25	35.30	36.41	36.21	35.36	35.36	32.85	24.82
26	83.20	88.88	76.67	86.11	80.21	69.73	73.68
27	50.00	50.77	51.73	48.15	44.58	60.87	39.90
28	56.50	57.93	56.92	57.31	53.43	44.06	62.27
29	35.50	20.85	30.46	33.68	39.28	35.10	34.13
30	36.10	34.92	34.59	36.03	35.78	32.35	16.05
31	20.90	42.96	15.75	33.59	35.93	44.32	53.03
32	51.00	60.39	61.63	54.00	54.33	50.00	54.81
33	95.20	79.24	92.32	97.05	80.43	71.92	56.78
34	28.00	30.20	29.90	27.95	29.14	26.68	22.74
35	60.00	56.15	57.96	60.36	57.60	53.10	30.99
36	46.80	45.49	45.32	44.13	45.88	34.48	35.04
37	39.30	35.34	35.41	37.21	37.24	38.70	18.93
38	38.00	38.96	36.98	39.21	43.23	25.48	23.56

**Table 7:** Performance comparison of previous works and the proposed model.

Reference	Model	Performance (R^2^)
Iftikhar et al. [57]	GEP	0.9670
	RFR	0.9130
Amin et al. [79]	DT	0.8900
	BgR	0.9200
	ADB	0.9100
This paper	CMRSA–ANN	0.9709

Note: GEP, gene expression programming; RFR, random forest regression; DT, decision trees; BgR, bagging regressors; ADB, AdaBoost regressors.

## Data Availability

The data used in this study are from published research: Iftikhar et al. [57].

## Supplementary Materials

The following supporting information can be downloaded at: https://www.mdpi.com/article/10.3390/ma16083135/s1.
