# Supplementary material for: Compressive Strength Prediction of Rice Husk Ash Concrete Using a Hybrid Artificial Neural Network Model
Source: Materials (Basel). 2023 Apr 16;16(8):3135. doi: 10.3390/ma16083135 (PMC10145703; doi:10.3390/ma16083135)
Supplement: Supplementary file 1 [file materials-16-03135-s001.zip › materials-2344030-supplementary.pdf]

Article

Compressive Strength Prediction of Rice Husk Ash Concrete Using a Hybrid Artificial Neural Network Model

Chuanqi Li [1,*], Xiancheng Mei [2], Daniel Dias [1], Zhen Cui [2] and Jian Zhou [3,*]

[1] Laboratory 3SR, CNRS UMR 5521, Grenoble Alpes University, 38000 Grenoble, France; daniel.dias@univ-grenoble-alpes.fr
[2] Institute of Rock and Soil Mechanics, Chinese Academy of Sciences, Wuhan 430071, China; xcmei@whrsm.ac.cn (X.M.); zcui@whrsm.ac.cn (Z.C.)
[3] School of Resources and Safety Engineering, Central South University, Changsha 410083, China
* Correspondence: chuanqi.li@univ-grenoble-alpes.fr (C.L.); j.zhou@csu.edu.cn (J.Z.)

No.	Age (days)	Cement (kg/m3)	Rice husk ash (kg/m3)	Water (kg/m3)	Super plasticizer (kg/m3)	Aggregate (kg/m3)	Experimental compressive strength (Mpa)
1	1	495	55	165	5.8	1819	22.7
2	1	500	0	160	5.5	1891	20.9
3	1	400	100	160	6.22	1859	22
4	1	425	75	170	5	1843	19.7
5	1	495	55	165	6.8	1819	34.7
6	1	500	0	160	6.5	1891	37.8
7	1	400	100	160	7.36	1859	34.2
8	1	425	75	170	6.4	1843	32.6
9	3	495	55	165	5.8	1819	47.9
10	3	500	0	160	5.5	1891	41.3
11	3	400	100	160	6.22	1859	48.7
12	3	425	75	170	5	1843	45.2
13	3	378	42	189	0	1810	17.6
14	3	495	55	165	6.8	1819	60.8
15	3	500	0	160	6.5	1891	63.9
16	3	400	100	160	7.36	1859	60.7
17	3	425	75	170	6.4	1843	57.3
18	7	495	55	165	5.8	1819	60.6
19	7	500	0	160	5.5	1891	51
20	7	400	100	160	6.22	1859	61.8
21	7	375	0	150	0	1970	30
22	7	356.25	18.75	142.5	0	1970	31.5
23	7	337.5	37.5	135	0	1970	32.5
24	7	318.75	56.25	127.5	0	1970	35.5
25	7	300	75	120	0	1970	31
26	7	364	19	203	0	1725	27.6
27	7	306	77	203	0	1725	29.7
28	7	249	134	203	0	1725	25.7

29	7	425	75	170	5	1843	57.6
30	7	495	55	165	6.8	1819	83.6
31	7	391	29	189	0	1810	32.4
32	7	500	0	160	6.5	1891	76.4
33	7	400	100	160	7.36	1859	82.8
34	7	425	75	170	6.4	1843	79.2
35	14	345	38	203	0	1725	35.3
36	14	287	96	203	0	1725	36.1
37	28	495	55	165	5.8	1819	72.8
38	28	500	0	160	5.5	1891	59.6
39	28	400	100	160	6.22	1859	72.7
40	28	375	0	150	0	1970	44.5
41	28	356.25	18.75	142.5	0	1970	45.5
42	28	337.5	37.5	135	0	1970	49.5
43	28	318.75	56.25	127.5	0	1970	50
44	28	300	75	120	0	1970	43
45	28	383	0	203	0	1725	37.1
46	28	326	57	203	0	1725	41.8
47	28	268	115	203	0	1725	37.6
48	28	425	75	170	5	1843	67.2
49	28	495	55	165	6.8	1819	95.2
50	28	500	0	160	6.5	1891	85.7
51	28	400	100	160	7.36	1859	94.3
52	28	420	0	189	0	1810	40.3
53	28	357	63	189	0	1810	46.9
54	28	425	75	170	6.4	1843	90.3
55	56	375	0	150	0	1970	51.5
56	56	356.25	18.75	142.5	0	1970	53.5
57	56	337.5	37.5	135	0	1970	56
58	56	318.75	56.25	127.5	0	1970	59.5
59	56	300	75	120	0	1970	52
60	90	495	55	165	5.8	1819	83.2
61	90	500	0	160	5.5	1891	66.8
62	90	400	100	160	6.22	1859	82.2
63	90	425	75	170	5	1843	75.8
64	90	364	19	203	0	1725	43.3
65	90	306	77	203	0	1725	46
66	90	249	134	203	0	1725	37.2
67	90	375	0	150	0	1970	55.5
68	90	356.25	18.75	142.5	0	1970	56.5
69	90	337.5	37.5	135	0	1970	63
70	90	318.75	56.25	127.5	0	1970	64

71	90	300	75	120	0	1970	61
72	90	378	42	189	0	1810	59
73	90	495	55	165	6.8	1819	104.1
74	90	500	0	160	6.5	1891	94
75	90	400	100	160	7.36	1859	103.3
76	90	425	75	170	6.4	1843	99.1
77	7	481	48.1	169.312	3.367	1040	39.5
78	7	427	85.4	163.968	3.416	1040	30.5
79	7	416	41.6	183.04	1.1232	1041	29.7
80	7	370	74	177.6	1.85	1041	23.6
81	7	367	36.7	201.85	1.101	1041	22.7
82	7	327	65.4	196.2	1.308	1041	20.8
83	28	481	48.1	169.312	3.367	1040	51.4
84	28	427	85.4	163.968	3.416	1040	47.4
85	28	416	41.6	183.04	1.1232	1041	40.8
86	28	370	74	177.6	1.85	1041	39.4
87	28	367	36.7	201.85	1.101	1041	34.5
88	28	327	65.4	196.2	1.308	1041	35.9
89	90	481	48.1	169.312	3.367	1040	64.5
90	90	427	85.4	163.968	3.416	1040	68.5
91	90	416	41.6	183.04	1.1232	1041	51.5
92	90	370	74	177.6	1.85	1041	57.3
93	90	367	36.7	201.85	1.101	1041	44.4
94	90	327	65.4	196.2	1.308	1041	52.9
95	1	450	0	238	11.25	1405	31.5
96	1	427.5	21.375	238	10.6875	1405	32.1
97	1	405	40.5	238	10.125	1405	33.3
98	1	382.5	57.375	238	9.5625	1405	34.5
99	1	360	72	238	9	1405	33.6
100	1	337.5	84.375	238	8.4375	1405	29.3
101	1	315	94.5	238	7.875	1405	29
102	28	450	0	238	11.25	1405	41.7
103	28	427.5	21.375	238	10.6875	1405	42.7
104	28	405	40.5	238	10.125	1405	44.2
105	28	382.5	57.375	238	9.5625	1405	46.8
106	28	360	72	238	9	1405	43.5
107	28	337.5	84.375	238	8.4375	1405	39.5
108	28	315	94.5	238	7.875	1405	38.2
109	56	450	0	238	11.25	1405	49.1
110	56	427.5	21.375	238	10.6875	1405	50.2
111	56	405	40.5	238	10.125	1405	52.1
112	56	382.5	57.375	238	9.5625	1405	55.3

113	56	360	72	238	9	1405	55.2
114	56	337.5	84.375	238	8.4375	1405	47
115	56	315	94.5	238	7.875	1405	45.9
116	90	450	0	238	11.25	1405	52.6
117	90	427.5	21.375	238	10.6875	1405	54.9
118	90	405	40.5	238	10.125	1405	57.3
119	90	382.5	57.375	238	9.5625	1405	61.2
120	90	360	72	238	9	1405	55.5
121	90	337.5	84.375	238	8.4375	1405	51.9
122	90	315	94.5	238	7.875	1405	50.2
123	1	783	87	212	3.6	1277	41
124	1	571	0	219	1	1566	30
125	1	514	57	218	1.4	1541	27
126	1	457	114	216	2.6	1515	26
127	1	400	171	215	3.7	1490	19
128	1	383	42	221	0.3	1670	16
129	3	783	87	212	3.6	1277	59
130	3	571	0	219	1	1566	46
131	3	514	57	218	1.4	1541	41
132	3	457	114	216	2.6	1515	38
133	3	400	171	215	3.7	1490	32
134	3	383	42	221	0.3	1670	26
135	7	783	87	212	3.6	1277	62
136	7	571	0	219	1	1566	50
137	7	514	57	218	1.4	1541	47
138	7	457	114	216	2.6	1515	47
139	7	400	171	215	3.7	1490	43
140	7	383	42	221	0.3	1670	37
141	14	783	87	212	3.6	1277	63
142	14	571	0	219	1	1566	54
143	14	514	57	218	1.4	1541	52
144	14	457	114	216	2.6	1515	52
145	14	400	171	215	3.7	1490	51
146	14	383	42	221	0.3	1670	40
147	28	783	87	212	3.6	1277	66
148	28	571	0	219	1	1566	56
149	28	514	57	218	1.4	1541	61
150	28	457	114	216	2.6	1515	60
151	28	400	171	215	3.7	1490	54
152	28	383	42	221	0.3	1670	47
153	56	783	87	212	3.6	1277	69
154	56	571	0	219	1	1566	60

155	56	514	57	218	1.4	1541	62
156	56	457	114	216	2.6	1515	61
157	56	400	171	215	3.7	1490	60
158	56	383	42	221	0.3	1670	51
159	90	783	87	212	3.6	1277	74
160	90	571	0	219	1	1566	67
161	90	514	57	218	1.4	1541	67
162	90	457	114	216	2.6	1515	69
163	90	400	171	215	3.7	1490	64
164	90	383	42	221	0.3	1670	56
165	7	364	19	203	0	1725	27.6
166	7	345	38	203	0	1725	28
167	7	326	57	203	0	1725	29.3
168	7	306	77	203	0	1725	29.7
169	7	287	96	203	0	1725	28.7
170	7	268	115	203	0	1725	27.4
171	7	249	134	203	0	1725	25.7
172	14	364	19	203	0	1725	34.2
173	14	345	38	203	0	1725	35.3
174	14	326	57	203	0	1725	36
175	14	306	77	203	0	1725	39.3
176	14	287	96	203	0	1725	36.1
177	14	268	115	203	0	1725	33.5
178	14	249	134	203	0	1725	31.1
179	28	364	19	203	0	1725	40
180	28	345	38	203	0	1725	41.3
181	28	326	57	203	0	1725	41.8
182	28	306	77	203	0	1725	42.5
183	28	287	96	203	0	1725	38.8
184	28	268	115	203	0	1725	37.6
185	28	249	134	203	0	1725	35.1
186	90	364	19	203	0	1725	43.3
187	90	345	38	203	0	1725	44.8
188	90	326	57	203	0	1725	45.7
189	90	306	77	203	0	1725	46
190	90	287	96	203	0	1725	43
191	90	268	115	203	0	1725	38.7
192	90	249	134	203	0	1725	37.2
